# Oral behavior, dental, periodontal and microbiological findings in patients undergoing hemodialysis and after kidney transplantation

**DOI:** 10.1186/s12903-016-0274-0

**Published:** 2016-08-17

**Authors:** Gerhard Schmalz, Anne Kauffels, Otto Kollmar, Jan E. Slotta, Radovan Vasko, Gerhard A. Müller, Rainer Haak, Dirk Ziebolz

**Affiliations:** 1 Department of Cariology, Endodontology and Periodontology, University of Leipzig, Liebigstr. 10-14, D 04103 Leipzig, Germany; 2 Department of General, Visceral and Pediatric Surgery, University Medical Center, Goettingen, Germany; 3 Present address: Department of General and Visceral Surgery, HELIOS Dr. Horst Schmidt-Kliniken, Wiesbaden, Germany; 4 Present address: Department of General and Visceral Surgery, St. Ingbert County Hospital, St. Ingbert, Germany; 5 Department of Nephrology and Rheumatology, University Medical Center, Goettingen, Germany

**Keywords:** Dental care, Hemodialysis, Kidney transplantation, Oral health, Oral hygiene

## Abstract

**Background:**

Aim of this single center cross-sectional study was to investigate oral behavior, dental, periodontal and microbiological findings in patients undergoing hemodialysis (HD) and after kidney transplantation (KT).

**Methods:**

Patients undergoing HD for end-stage renal failure and after KT were investigated. Oral health behavior was recorded using a standardized questionnaire, e.g. dental behavior, tooth brushing, oral hygiene aids. Oral investigation included screening of oral mucosa, dental findings (DMF-T) and periodontal situation (Papilla bleeding index [PBI] periodontal probing depth [PPD] and clinical attachment loss [CAL]). Additionally, microbiological analysis of subgingival biofilm samples (PCR) was performed. Statistical analysis: Student’s *t*-test or Mann–Whitney-*U*-test, Fisher’s exact test (α = 5 %).

**Results:**

A total of 70 patients (HD: *n* = 35, KT: *n* = 35) with a mean age of 56.4 ± 11.1 (HD) and 55.8 ± 10.9 (KT) years were included. Lack in use of additional oral hygiene (dental floss, inter-dental brush) was found. KT group presented significantly more gingivial overgrowth (*p* = 0.01). DMF-T was 19.47 ± 5.84 (HD) and 17.61 ± 5.81 (KT; *p* = 0.21). Majority of patients had clinically moderate and severe periodontitis; showing a need for periodontal treatment of 57 % (HD) and 71 % (KT; *p* = 0.30). Significantly higher prevalence of *Parvimonas micra* and *Capnocytophaga species* in the HD group were found (*p* < 0.01).

**Conclusion:**

Periodontal treatment need and lack in oral behavior for both groups indicate the necessity of an improved early treatment and prevention of dental and periodontal disease, e.g. in form of special care programs. Regarding microbiological findings, no major differences between KT and HD patients were found.

## Background

When the functional capacity of the kidneys decreases below 5–10 % of the normal efficiency, renal replacement therapy, i.e. hemodialysis (HD), peritoneal dialysis, or kidney transplantation (KT) is necessary as a life-supporting measure [1, 2]. HD, which improves the long-term survival of patients with end stage kidney disease, is the most common form of renal replacement therapy [3]. Patients undergoing hemodialysis (HD) for chronic renal failure often suffer from different systemic changes such as general weakening and increased susceptibility toward infection [4]. Alongside with these systemic changes these patients often present high need for dental and periodontal treatment due to various reasons [4–6]. Hemodialysis and reduction of oral fluid intake can lead to reduced saliva and xerostomia resulting in changes of the oral mucous membranes, increased dental calculus formation and thus leading to risk for viral and fungal infection [2, 7, 8]. In addition, a considerably high percentage of hemodialysis patients neglect oral hygiene measures, which is frequently caused by high burden of HD [9, 10]. Accordingly, oral health is often getting worse during HD therapy [5, 11, 12].

A further aspect is the fact, that HD patients are also candidates for KT [13]. Due to the immunosuppression, patients with organ transplantations are on a potentially higher risk in dental practice [14]. In order to avoid complications caused by dental interventions in transplant patients, a dental investigation before organ transplantation is recommended [15]. Furthermore, patients waiting for organ transplantation should be early dentally rehabilitated [13, 16]. Beside of that, the associated immunosuppressive therapy often leads to undesired oral effects, especially gingival overgrowth or oral infections with Candida species [17]. Consequently, a good maintenance is needed for transplant and HD patients to reduce oral inflammation [5, 11, 18, 19].

Considering these demands, it should be expected, that patients after KT show a better oral hygiene compared to patients under HD. Furthermore, both KT and HD patients should show better oral health behavior and status compared to general population. However, the current data show a reduced oral health behavior and status, especially for HD patients [20], what is contradictory to the demand of a sufficient maintenance of these patients. Very few data concerning this are available, especially for KT patients [20]. Accordingly, a recent meta-analysis concluded further investigations of these patients to be necessary [20]. Therefore, the current study examined patients with renal insufficiency undergoing hemodialysis and after kidney transplantation for their oral hygiene behavior, dental and periodontal status. Additionally, all patients were screened for periodontal pathogens. It was therefore aim of the current study to test the hypothesis that KT patients have better oral health behavior and status compared to HD patients.

## Methods

This clinical single center cross-sectional study was reviewed and approved by the ethics committee of the University Medical Center Goettingen, Germany (No. 43/9/07). All patients provided written informed consent and guidelines for ethical approvals for human subjects were followed in accordance with the Declaration of Helsinki.

### Patients

The included HD and KT patients are part of a dialysis and transplant study and were selected for this specific question using previously defined in- and exclusion as well as specific matching criteria. No preliminary power calculation was performed. The patient group size was determined by the number of patients after KT attending the Department of General, Visceral and Pediatric Surgery of the University Medical Center Goettingen (transplant center) from February to July 2012. Patients undergoing HD for end-stage renal failure attending the Department of Nephrology of the University Medical Center Goettingen were matched according to age, gender, smoking habits and diabetes status of the KT group, if possible.

HD group: Patients who before had been registered on the Eurotransplant waiting list for KT were asked to voluntarily participate in the current study. The following exclusion criteria were defined: patients <18 years, presence of an additional infectious disease (HIV or TBC infection), seizure or nervous disorder and inability to undergo oral examination.

KT group: Patients having undergone KT irrespective to the time span since transplantation were asked to take part in the current study in the context of a regular/routine subsequent appointment at the transplantation outpatient clinic of the University Medical Center Goettingen. Exclusion criteria were equal in both groups. Patients were assigned to one group only. Patients with dental or periodontal treatment need were not treated, but informed about the necessity of treatment.

### Patient questionnaire

Both groups of patients were asked to fill out a standardized questionnaire regarding their general anamnesis (general illnesses, general medication, reason for dialysis and/or transplantation, especially for KT: date of transplantation and current immunosuppressive therapy). In a special dental questionnaire patients were interrogated whether information about the association between oral health and dialysis/transplantation had been provided and a dental check-up or comprehensive dental treatment had taken place before transplantation (during HD time) or registration on the Eurotransplant waiting list (yes/no, when). In addition, patients were questioned about their personal oral hygiene behavior (tooth brushing, additional oral hygiene aids), and their habit of visiting their dentist, i.e. regular dental check-up or only in case of complaints. The questionnaires were designed in accordance to previous studies by this working group [18, 21].

### Dental examination

All patients were examined under standardized conditions in a dental unit with light using mirror and probe. The examination was performed by a skilled dentist in the Department of Preventive Dentistry, Periodontology and Cariology, University Medical Center Goettingen. Investigation included inspection of the oral mucous membranes, dental findings and evaluation of the periodontal situation.

#### Inspection of the oral mucous membranes

At the beginning of the examination, the oral mucous membranes were examined visually to detect existing gingival overgrowth (yes or no).

#### Dental findings (DMF-T)

The DMF-T was assessed visually with mirror and probe. Based on the number of decayed, missing, and filled teeth, the DMF-T index was determined: All teeth with a reasonable suspicion of/or definitely showing a cavity in the dentine layer was assigned to the D (= decayed) component, filled and crowned teeth were evaluated component F (=filled), missing teeth were assigned to the M (=missing) component. The DMF-T generally reflects the caries experience of the person examined. In addition, the degree of caries restoration (%) was calculated: ratio of filled teeth (FT) to the carious (DT) plus filled teeth (FT) (FT/(DT + FT) × 100) [22].

#### Periodontal situation

Assessment of gingival inflammation was performed with Papilla bleeding index (PBI) using a periodontal probe (PCP 15; Hu-Friedy, Chicago, IL, USA); PBI ranged from score 0 (no bleeding/inflammation-free gingiva) to 4 (profuse bleeding/severe inflammation) [23]. To investigate periodontal situation a periodontal status was executed, including periodontal probing depth (PPD) and bleeding on probing (BOP: positive) as well as clinical attachment loss (CAL) at 6 measurement points per tooth using a millimeter-scaled periodontal probe (PCP 15; Hu-Friedy, Chicago, IL, USA). According to the definition of Page and Eke [24] periodontitis was determined in three categories: 1) severe periodontitis, 2) moderate periodontitis or 3) no/mild periodontitis [24]. The periodontal treatment need was determined by the results of the periodontal status according the periodontal screening index (PSR®/PSI); periodontal treatment need: PSR®/PSI scores of 3 (PPD: 3.5–5.5 mm) and 4 (PPD: > 5,5 mm) [25–27].

### Microbiological analysis

After removal of supragingival plaque from ≥2 teeth (maxilla and mandible) up to a maximum of four teeth (first to fourth quadrant) with the deepest periodontal pockets, subgingival plaque biofilm samples were taken using sterile paper tips (10 s) and pooled. To avoid contamination with saliva, cotton rolls were placed before sample collection. Paper points, which were contaminated with blood, were discarded. Microbiological analysis of the periodontal pathogens was carried out using polymerase chain reaction analysis (PCR) in the clinical laboratory of the Department of Preventive Dentistry, Periodontology and Cariology, University Medical Center Goettingen. For the semiquantitative detection of the bacterial colonization of the patients’ oral samples a commercial test system was used (Micro-IDentplus®-Test, HainLifescience, Nehren, Germany) according to the manufacturers protocol. The amplification was executed using a 35-μl mixture of primers and dNTPs (Hain Lifescience, Nehren, Germany), 10.5 μl Mastermix (Qiagen, Hilden, Germany) and 5 μl of the DNA sample or 5 μl of water as a negative control. Amplification cycles were performed in a thermo cycler (Biometra, Goettingen, Germany). The hybridization was executed in accordance to Micro-IDent plus protocol in a TwinCubator (Hain Lifescience, Nehren, Germany).

With this analysis, the following 11 different periodontal pathogenic bacteria were detected: *Aggregatibacter actinomycetemcomitans* (*Aa, detection threshold >10*^*2*^)*, Porphyromonas gingivalis (Pg), Tannerella forsythia (Tf), Treponema denticola (Td), Prevotella intermedia (Pi), Parvimonas micra (PM), Fusobacterium nucleatum (Fn), Campylobacter rectus (Cr), Eubacterium nodatum (En), Eikenalla corrodens (Ec), Capnocytophaga species(Cs);* detection threshold >10^3^).

### Statistical analysis

Data were entered prospectively in a Microsoft Excel-based database (Microsoft Corporation, Redmont, WA, USA). Statistical analyses were performed using the statistical software package SPSS Statistics 21® (IBM, Chicago, USA). Data are presented as absolute numbers, mean value (MV) ± standard deviation (SD), or percentage unless indicated otherwise. For quantitative data, comparison of mean values was performed using Student’s *t*-test or Mann–Whitney-*U*-test, depending on normal distribution of the data, respectively. Categorical variables were analyzed by Fisher’s exact test. A *p*-value 0.05 was considered statistically significant.

## Results

### Patients

A total of 70 patients (HD: *n* = 35, KT: *n* = 35) with a mean age of was 56.4 ± 11.1 (HD) and 55.8 ± 10.9 (KT) years were included in the current study. All demographic data, underlying kidney disease for dialysis/transplantation and immunosuppresive medication are listed in Table 1.

**Table 1:** Characteristics of patients undergoing hemodialysis (HD, *n* = 35) and after kidney transplantation (KT, *n* = 35). Data are given as %(n) or mean ± standard deviation, [range]

		HD group	KT group	*p*-value
Gender (female)		40 % (14)	43 % (15)	>0.05
Age in years		56.4 ± 11.1 (29–79)	55.8 ± 10.9 (35–78)	>0.05
Smoking habits	Smoker	17.6 % (6/34)	10 % (3/30)	>0.05
	Non-smoker	82.4 % (28/34)	90 % (27/30)	
Causal underlying disease	Polycystic kidney	8.6 % (3)	11.4 % (4)	>0.05
	Unknown	5.7 % (2)	22.9 % (8)	
	Glomerulonephritis	28.6 % (10)	51.4 % (18)	
	Diabetic nephropathy	14.3 % (5)	2.9 % (1)	
	Others	42.8 % (15)	11.4 % (4)	
Co-morbidities	Diabetes	14.3 % (5)	14.3 % (5)	>0.05
	CHD	62.9 % (22)	28.6 % (10)	
	Arterial hypertension	94.3 % (33)	74.3 % (26)	
	Pulmonary disease	28.6 % (10)	14.3 % (5)	
	Tumor	25.7 % (9)	8.6 % (3)	
	Osteoporosis	11.4 % (4)	17.1 % (6)	
Time after KT	Years	-	14.1 ± 7.1 [4–30]	-
	>1 to 5 years		11.4 % (4)	
	>5 years		88.4 % (31)	
Time undergoing HD	Years	5.5 ± 6.4 [1–36]	-	-
	<1 year	13.3 %		
	>1 to 5 years	38.2 % (13)		
	>5 years	35.3 % (12)		
Immunosuppressive medication	Tacrolimus (Prograf)	-	11 % (4)	-
	Tacrolimus (Advagraf)		26 % (9)	
	Cyclosporin A		34 % (12)	
	MMF (Cellcept)		23 % (8)	
	MMF (Myfortic)		26 % (9)	
	GC (Prednisolon)		43 % (15)	

Significance level < 0.05

*CHD* coronary heart disease, *MMF* Mycophenolat mofetil, *GC* glucocorticoide, *KT* kidney transplantation, *HD* hemodialysis

### Patient questionnaire

Results of the patient questionnaire concerning dental check-ups and oral hygiene behavior are given in Table 2. The number of returned answers is also displayed in Table 2 as not every patient answered each question. Majority of patients were found to visit the dentist for regular dental check-up (HD: 85.7 % (30/35), KT: 93.3 % (28/30), *p* > 0.05). Of the KT patients, 69 % (20/29) stated to had dental treatment before transplantation, and 68 % (21/31) answered to know about the necissity of antibiotical prophylaxes for dental treatment. In performing oral hygiene lack in usage of dental floss/IDR-brush (HD: 31.4 % (11/35) KT: 50 % (15/30), *p* > 0.05) and fluoride gel (HD: 5.7 % (2/35), KT: 20 % (6/30), *p* > 0.05) was found. There were no significant differences between groups (*p* > 0.05).

**Table 2:** Results of the patients’ questionnaire. Data are given as %(n)

		HD group	KT group	*p*-value
Regular contact with a dentist		77.1 % (27/35)	83.3 % (25/30)	>0.05
Reason for visiting dentist	Regular check-up	85.7 % (30/35)	93.3 % (28/30)	>0.05
	Complaint	14.3 % (5/35)	6.7 % (2/30)	
Last dental examination	0–3 months	28.6 % (10/35)	40 % (12/30)	>0.05
	3–12 months	54.3 % (19/35)	53.3 % (16/30)	
	>12 months	17.1 % (6/35)	6.7 % (2/30)	
Dental treatment before KT		-	69 % (20/29)	-
Information about necessity of antibiotical prophylaxis		-	68 % (21/31)	-
Oral hygiene: tooth brushing	<1×/day	2.9 % (1/35)	0.0 % (0/27)	>0.05
	1–2×/day	88.7 % (30/35)	85.2 % (23/27)	
	>2×/day	11.4 % (4/35)	14.8 % (4/27)	
Oral hygiene aids	Manual toothbrush	85.7 % (30/35)	62.9 % (22/35)	>0.05
	Power toothbrush	31.4 % (11/35)	33.3 % (10/35)	
	Dental floss / inter-dental brush	31.4 % (11/35)	50 % (15/30)	
	Mouth rinse	48.6 % (17/35)	50 % (15/30)	
	Fluoride gel	5.7 % (2/35)	20 % (6/30)	

Significance level < 0.05

*KT* kidney transplantation, *HD* hemodialysis

### Dental examination

#### Inspection of the oral mucous membranes

Patients in the KT group were found to present significantly more gingivial overgrowth (HD: 0 % (0/35), KT: 20 % (7/35), *p* = 0.01).

#### Dental findings (DMF-T)

Dental findings of patients in both groups are shown in Table 3. Five HD and 2 KT patients were toothless (*p* = 0.43). A significant difference comparing DMF-T of both groups could not be found (HD: 19.47 ± 5.84, KT: 17.61 ± 5.81, *p* = 0.21).

**Table 3:** Comparison of the oral health parameters in patients undergoing hemodialysis (HD, *n* = 35) and after kidney transplantation (KT, *n* = 35). Data are given as %(n) or mean ± standard deviation, [range]

Oral health parameters		HD group	KT group	*p*-value
Gingivial overgrowth		0 % (0/35)	20 % (7/35)	0.01
Edentulous patients		14.3 % (5/35)	5.7 % (2/35)	0.43
DMF-T patients with teeth		19.47 ± 5.84 [4–28]	17.61 ± 5.81 [8–28]	0.21
D-T patients with teeth		1.13 ± 1.68 [0–6]	0.58 ± 1.15 [0–6]	0.13
M-T patients with teeth		9.67 ± 8.90 [1–27]	7.15 ± 6.69 [0–27]	0.21
F-T patients with teeth		8.67 ± 5.56 [1–18]	9.88 ± 4.46 [1–19]	0.34
Degree of caries restoration		89.76 ± 17.97 % [33.3–100 %]	94.29 ± 12.61 % [33.3–100 %]	0.23
Gingivial inflammation (PBI)		0.38 ± 0.27 [0–1.00]	0.52 ± 0.49 [0–1.62]	0.17
Periodontitis	No/mild	6.7 % (2/30)	20.6 % (7/34)	0.16
	Moderate	40 % (12/30)	47.1 % (16/34)	0.62
	Severe	53.3 % (16/30)	32.4 % (11/34)	0.13
Need for periodontal treatment		56.7 % (17/30)	71.4 % (25/35)	0.30

Significance level < 0.05

*DMF-T* number of carious, missing and filled teeth (caries index), *D-T* carious teeth, *M-T* missing teeth, *F-T* filled teeth, *PBI* papillary bleeding index, *KT* kidney transplantation, *HD* hemodialysis

#### Periodontal situation

Periodontal findings were not significantly different comparing both groups. Majority of patients had clinically moderate (HD: 40 %, KT: 47 %; *p* = 0.62) and severe periodontitis (HD: 53 %, KT: 32 %; *p* = 0.13), showing a need for periodontal treatment of 57 % (HD) and 71 % (KT; *p* = 0.3; Table 3).

### Microbiological analysis

**Fig. 1:**
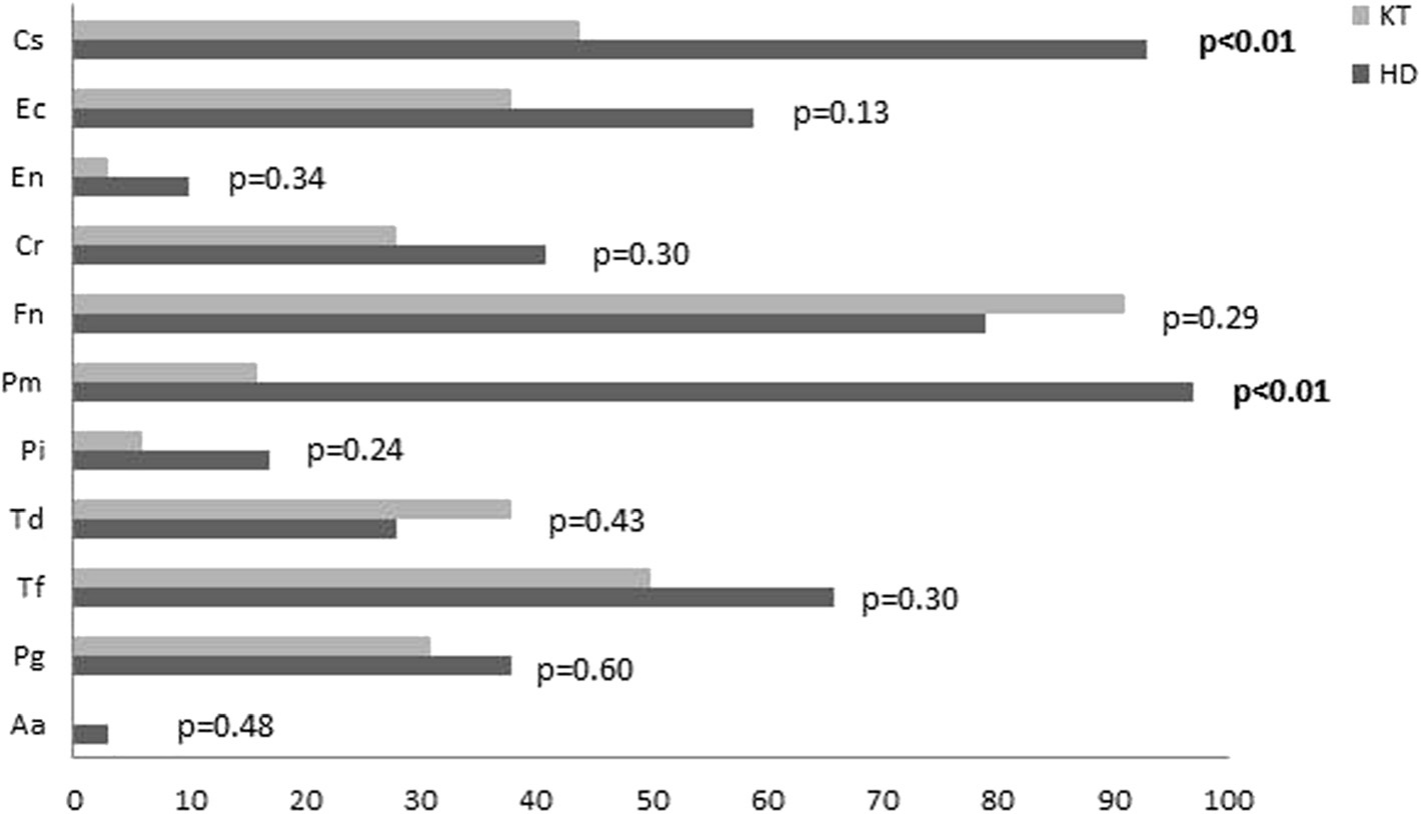
Prevalence of periodontal pathogenic bacteria in patients undergoing hemodialysis (HD, *n* = 29) and after kidney transplantation (KT, *n* = 32). Data are given in %. Aa: *Aggregatibacter actinomycetemcomitans (*detection threshold: >10^2^)*,* Pg: *Porphyromonas gingivalis,* Tf: *Tanerella forsythia,* Td: *Treponema denticola,* Pi: *Prevotella intermedia*, Pm: *Parvimonas micra,* Fn: *Fusobacterium nucleatum,* Cr: *Campylobacter rectus*, En: *Eubacterium nodatum,* Ec: *Eikanella corrodens* and Cs: *Capnocytophaga spec.;* detection threshold: >10^3^; KT: kidney transplantation, HD: hemodialysis; significance level < 0.05, significant results are given in bold

Prevalence of the different bacteria was similar in both groups (Table 4), but with significantly higher prevalence of *Pm* (HD: 97 %, KT: 16 %) and *Cs* (HD: 93 %, KT: 44 %) in the HD group (*p* < 0.01; Fig. 1).

**Table 4:** Dental findings (DMF-T) and periodontal findings (periodontal treatment need, PSR®/PSI) of the Fourth German Oral Health Study (DMS IV) and the present study. Data are given as % or mean ± standard deviation

Oral finding		DMS IV		Present study	
		Age group: 35 – 44 years	Age group 65 – 74 years	HD group 56.4 years	KT group 55.7 years
DMF-T		14.5 ± 5.7	22.1 ± 5.9	19.5 ± 5.8	17.6 ± 5.8
D-T		0.5	0.3	1.1 ± 1.7	0.6 ± 1.2
M-T		2.4	14.1	9.7 ± 8.9	7.2 ± 6.7
F-T		11.7	7.7	8.7 ± 5.6	9.9 ± 4.5
Prevalence of periodontitis	No/mild	27 %	12 %	7 %	21 %
	Moderate	53 %	48 %	40 %	47 %
	Severe	20 %	40 %	53 %	32 %
Periodontal treatment need [PSR®/PSI]	No [score 0–2]	26.5 %	12 %	43 %	29 %
	Yes [score 3–4]	73.5 %	88 %	57 %	71 %

*DMF-T* number of carious, missing and filled teeth (caries index), *D-T* carious teeth, *M-T* missing teeth, *F-T* filled teeth, *PSR®/PSI* Periodontal Screening Index, *KT* kidney transplantation, *HD* hemodialysis

## Discussion

Summary of the main results: Although most patients (HD: 77.1 %, KT: 83.3 %) stated to be in regular contact with a dentist, high need for periodontal treatment (HD: 56.7 %, KT: 71.4 %; *p* = 0.30) was found. Gingival overgrowth was only detected in KT group. Subsequently, no significant differences in dental examination (DMF-T) and severity of periodontitis was shown between groups. Furthermore, a significantly higher prevalence of *Pm* and *Cs* in the HD group was found (*p* < 0.01).

Comparison with existing literature: it must be considered the fact that this is, to the best of author’s knowledge, the first study comparing HD and KT patients regarding oral health behavior, dental and periodontal as well as microbiological findings. Accordingly, an interpretation considering the recent literature is difficult. Taking into account no healthy control was investigated, results of dental and periodontal examination can be discussed in relation to Fourth German Oral Health study (DMS IV) by Micheelis and Schiffner 2006, a representative study for German population [28].

Looking at the dental behavior, a previous study of this working group presented similar results regarding oral hygiene for HD patients in Germany, but showed that only the half of patients visit dentist in case of problems [21]. A recent meta-analysis by Rouspo et al. demonstrated worse results in oral hygiene behavior for HD patients compared to the current studies results. Especially regarding use of dental floss Ruospo et al. showed values of 11.4 %, which was three fold higher in the current study (HD: 31.4 %). Furthermore, while the current study demonstrated only 2.9 % of HD patients to brush their teeth <1×/day, the meta-analysis showed 25.5 % to never brush their teeth [20]. It must be taken into account that the studies included in the analysis were executed in different countries with different hygienic standards, which might explain the large differences. Therefore, a multinational cohort study of HD patients showed 91.5 % of patients to brush their teeth daily, but also only 8.2 % using dental floss [29]. While the current study did not show an increased dental and oral hygiene behavior, results are not available for KT patients in literature. An increased oral health behavior is therefore not found for HD and KT patients.

Regarding gingival overgrowth, the higher prevalence in KT patients is confirmed by recent literature [19, 30, 31], resulting in a high need for good oral hygiene, because deficiencies regarding this issue decisively encourage gingival overgrowth [19]. It must be considered, that gingival overgrowth is a multifactorial process with different influencing factors, e.g. age, gender, genetic predisposition, additional medications and oral hygiene status [32].

Dental findings showed a DMF-T of on average 19.47 (HD) and 17.61 (KT). In DMS IV, similar DMF-T values were demonstrated for patients with an age of 35–44 years (14.5) and of 65–74 years (22.1; Table 4) [28]. The mean age in the current study was 56.4 ± 11.1 years, so the values of DMF-T for HD and KT patients in the current study are lying between DMS IV values and age groups and thus appear not different compared to German population. The previous study of this working group showed a higher DMF-T with an average 22.1 ± 6.5 for HD patients. However, patients were at a higher mean age (63.9 ± 13.0 years) in that study [18]. In international literature, DMF-T values show a high diversity in HD patients, with ranges between 6.6 and 26 [20]. This might be caused by different populations, mean ages and study designs as well. A recent multinational cohort study showed a mean DMF-T of 19.3, which is similar to the current study [29]. Only few results are available for KT patients, so Vesterinen et al. showed a DMF-T of 25.7, which is almost higher compared to the current study [33]. In contrast, Bots et al. found lower values of 15.5 for KT patients, and showed no significant differences in dental findings between KT and HD patients, what corresponds to the findings of the current study [34].

Periodontal findings demonstrated high periodontal treatment need in both groups (HD: 57 %, KT: 71 %). Compared to DMS IV results (35–44 years: 74 %, 65–74 years: 88 %) these findings were slightly lower (Table 4) [28]. This is in accordance to findings for HD patients of comparable studies, however they showed periodontal situation becoming worse with duration of dialysis therapy [4, 11, 12]. Furthermore, the mean prevalence of periodontitis in HD patients in Europe was reported to be 67.7 %, which is lower than in the current study (HD: 93 %, KT: 80 %) [20]. Bots et al. found no statistically significant differences in periodontal situation between HD and KT patients over a period of two years [34]. Further available studies for KT patients are rare. In this context, the immunosuppressive therapy might have an influence on periodontal inflammation [35]. This issue was not investigated in the current study. Nevertheless, lack of periodontal health might lead to complications for KT patients [36].

Taking into account, that HD patients are also candidates for transplantation [13], recommendations and a good maintenance is need for transplant and HD patients to reduce periodontal inflammation [5, 11, 18, 19]. Albeit periodontal treatment need of HD and KT patients in the current study was slightly lower than in DMS IV results for German population, clearly lower treatment need should be given, especially for KT patients. It is therefore necessary to mention, that not the same patients were investigated before and after KT and mean time after KT was 14.1 years in the current study. Because of 69 % of KT patients had dental treatment before transplantation, one might expect a better oral health after transplantation, however, in this case a lack of maintenance is apparent.

Microbiological analysis showed significantly higher prevalence of *Pm* and *Cs* in the HD group. An explanation could be a change in the subgingival microflora caused by uremia, resulting in increased bacterial growth [37]. However, there is no founded hypothesis, why in the current study uremia should cause especially higher prevalence of *Pm* and *Cs* in HD group. Higher prevalence of periodontal pathogenic bacteria were detected in HD patients compared to healthy controls in several studies, however, findings of the current study are not confirmed [37–39]. Results for KT showed fewer putative anaerobic pathogens in periodontal pocket of immunosuppressed KT patients [40]; the current study confirms this for *Pm* and *Cs*. Furthermore Leung et al. concluded a subgingival alteration [41]. Comparative results between HD and KT are not available, what makes an interpretation difficult, but an influence of KT and immunosuppression on subgingival bacteria appears to be possible. Accordingly, the clinical relevance of the current study’s findings is not clear. It is questionable, if it has an influence on clinical and periodontal alteration or resistance to periodontal treatment.

### Strengths and limitations

To the best of author’s knowledge, this is the first study, comparing HD and KT patients regarding oral behavior, dental, periodontal and microbiological findings. The waiver of a healthy control group is a limitation of the current study. Finding a healthy control group for the population in the current study would have been difficult, and a comprehensive study of general German population (DMS IV) serves as a good reference to discuss the results as well. Previous studies of this working group also used DMS IV results for discussion of their results [18, 21]. Nevertheless, for microbiological findings a healthy control group would help to correctly interpret the findings. A further limitation is the fact that it was not possible to investigate same patients before and after KT. Additionally, the time span after KT might affect the oral health status and behavior. For a stronger statement, time span after KT could have been accounted. However, it was chosen to include all recruited patients in order to reach a preferably large group. In addition, the examination of influence of specific immunosuppressive therapy on clinical and microbiological findings would be interesting. However, because of the heterogeneity of the medication used, the sample size for the different immunosuppressive medications would be very small, and therefore not very meaningful. These questions are specific and should be subject of future studies. The current study serves as a first overview on potential differences between HD and KT patients and presents results as required by Ruospo et al. [20].

## Conclusion

Within the limitations of the study, dental and periodontal health is not different between HD and KT patients and appears to be not clearly better than for general population. Neither HD nor KT patients seem to have an increased oral health behavior, although this is suggested in literature and based on the current study’s findings. Consequently, an improved early treatment and prevention of dental and periodontal disease, e.g. in form of special care programs is needed. Regarding microbiological findings, no major differences between KT and HD patients were found.

## Abbreviations

BOP: Bleeding on probing
CAL: Clinical attachment loss
DMF-T: Decayed (D) missing (M), and filled (F) teeth (T) index
HD: Hemodialysis
HIV: Human immunodeficiency virus
KT: Kidney transplantation
PBI: Papilla bleeding index
PCR: Polymerase chain reaction analysis
PPD: Periodontal probing depth
PSR®/PSI: Periodontal screening index
TBC: Tuberculosis

## References

[1] Bots CP, Poorterman JHG, Brand HS (2006). The oral health status of dentate patients with chronic renal failure undergoing dialysis therapy. Oral Dis.

[2] Jover Cerveró A, Bagán JV, Jiménez SY, Poveda Roda R (2008). Dental management in renal failure: patients on dialysis. Med Oral Patol Oral Cir Bucal.

[3] Himmelfarb J, Ikizler TA (2010). Hemodialysis. N Engl J Med.

[4] Bayraktar G, Kurtulus I, Kazancioglu R (2008). Evaluation of periodontal parameters in patients undergoing peritoneal dialysis or hemodialysis. Oral Dis.

[5] Cengiz MI, Sümer P, Cengiz S, Yavuz U (2009). The effect of the duration of the dialysis in hemodialysis patients on dental and periodontal findings. Oral Dis.

[6] Klassen JT, Krasko BM (2002). The dental health status of dialysis patients. J Can Dent Assoc.

[7] Kaushik A, Reddy SS, Umesh L, Devi BKY, Santana N, Rakesh N (2013). Oral and salivary changes among renal patients undergoing hemodialysis: a cross-sectional study. Indian J Nephrol.

[8] Swapna LA, Reddy RS, Ramesh T (2013). Oral health status in haemodialysis patients. J Clin Diagn Res.

[9] Borawski J, Wilczyńska-Borawska M, Stokowska W, Myśliwiec M (2007). The periodontal status of pre-dialysis chronic kidney disease and maintenance dialysis patients. Nephrol Dial.

[10] Grubbs V, Plantinga LC, Crews DC (2011). Vulnerable populations and the association between periodontal and chronic kidney disease. Clin J Am Soc Nephrol.

[11] Bayraktar G, Kurtulus I, Duraduryan A (2007). Dental and periodontal findings in hemodialysis patients. Oral Dis.

[12] de Souza CM, Braosi AP, Luczyszyn SM (2014). Association among oral health parameters, periodontitis, and its treatment and mortality in patients undergoing hemodialysis. J Periodontol.

[13] Guggenheimer J, Mayher D, Eghtesad B (2005). A survey of dental care protocols among US transplant centers. Clin Transplant.

[14] Maestre-Vera JR, Gómez-Lus Centelles ML (2006). Antimicrobial prophylaxis in oral surgery and dental procedures. Med Oral Cir Bucal.

[15] Guggenheimer J, Eghtesad B, Stock DJ (2003). Dental management of the (solid) organ transplant patient. Oral Surg Oral Med Oral Pathol Oral Radiol Endod.

[16] Melkos AB, Massenkeil G, Neuhaus R, Hummel M, Arnold R, Reichart PA (2005). Organ transplantation-assessment of dental procedures. Oral Biosci Med.

[17] King GN, Healy CM, Glover MT (1994). Prevalence and risk factors associated with leukoplakia, hairy leukoplakia, erythematous candidiasis and gingival hyperplasia in renal transplant patients. Oral Surg Oral Med Oral Pathol.

[18] Ziebolz D, Hraský V, Goralczyk A, Hornecker E, Obed A, Mausberg RF (2011). Dental care and oral health in solid organ transplant recipients: a single center cross-sectional study and survey of German transplant centers. Transpl Int.

[19] Kaswan S, Patil S, Maheshwari S, Wadhawan R (2015). Prevalence of oral lesions in kidney transplant patients: a single center experience. Saudi J Kidney Dis Transpl.

[20] Ruospo M, Palmer SC, Craig JC (2014). Prevalence and severity of oral disease in adults with chronic kidney disease: a systematic review of observational studies. Nephrol Dial Transplant.

[21] Ziebolz D, Fischer P, Hornecker E, Mausberg RF (2012). Oral health of hemodialysis patients: a cross-sectional study at two German dialysis centers. Hemodial Int.

[22] WHO (1997). World Health Organization: oral health surveys, basic methods 4^th^ edition.

[23] Lange DE, Plagmann HC, Eenboom A, Promesberger A (1977). Clinical methods for the objective evaluation of oral hygiene. Deutsch Zahnärztl Zeitschr.

[24] Page RC, Eke PI (2007). Case definitions for use in population-based surveillance of periodontitis. J Periodontol.

[25] Diamanti-Kipioti A, Papapanou TN, Moraitaki-Zamitsai A, Lindhe J, Mitsis F (1993). Comparative estimation of periodontal conditions by means of different index systems. J Clin Periodontol.

[26] Ainamo J, Barmes D, Beagrie G, Cutress T, Martin J, Sardo-Infirri J (1982). Development of the World Health Organization (WHO) Community Periodontal Index of Treatment Needs (CPITN). Int Dent J.

[27] Meyle J, Jepsen S (2000). The Periodontal Screening-Index (PSI). Parodontol.

[28] Micheelis W, Schiffner U (2006). The Fourth German Oral Health Study (DMS IV). Institut der Deutschen Zahnärzte (Hrsg.); (IDZ Materialienreihe Band 31).

[29] Palmer SC, Ruospo M, Wong G (2015). Dental health and mortality in people with end-stage kidney disease treated with hemodialysis: a multinational cohort study. Am J Kidney Dis.

[30] Dirschnabel AJ, Martins Ade S, Dantas SA (2011). Clinical oral findings in dialysis and kidney-transplant patients. Quintessence Int.

[31] Boratyńska M, Radwan-Oczko M, Falkiewicz K, Klinger M, Szyber P (2003). Gingival overgrowth in kidney transplant recipients treated with cyclosporine and its relationship with chronic graft nephropathy. Transplant Proc.

[32] Seymour RA, Ellis JS, Thomason JM (2000). Risk factors for drug-induced gingival overgrowth. J Clin Periodontol.

[33] Vesterinen M, Ruokonen H, Leivo T (2007). Oral health and dental treatment of patients with renal disease. Quintessence Int.

[34] Bots CP, Brand HS, Poorterman JH (2007). Oral and salivary changes in patients with end stage renal disease (ESRD): a two year follow-up study. Br Dent J.

[35] Pereira-Lopes O, Sampaio-Maia B, Sampaio S (2013). Periodontal inflammation in renal transplant recipients receiving everolimus or tacrolimus - preliminary results. Oral Dis.

[36] Zwiech R, Bruzda-Zwiech A (2013). Does oral health contribute to post-transplant complications in kidney allograft recipients?. Acta Odontol Scand.

[37] Bastos JA, Diniz CG, Bastos MG (2011). Identification of periodontal pathogens and severity of periodontitis in patients with and without chronic kidney disease. Arch Oral Biol.

[38] Paula H, Artese C (2012). Effect of non-surgical periodontal treatment on the subgingival microbiota of patients with chronic kidney disease. Braz Oral Res.

[39] Takeuchi Y, Ishikawa H, Inada M (2007). Study of the oral microbial flora in patients with renal disease. Nephrology (Carlton).

[40] Vieira ML, Martins WJ, Grisi MF, Novaes AB, Souza SL, Salvador SL (2002). Clinical and microbiological analysis of periodontally diseased sites after renal transplant. Spec Care Dentist.

[41] Leung WK, Yau JY, Jin LJ (2003). Subgingival microbiota of renal transplant recipients. Oral Microbiol Immunol.

